# Dendritic Cell Vaccination, Immune Regulation, and Clinical Outcomes in Ovarian Cancer

**DOI:** 10.3389/fimmu.2013.00382

**Published:** 2013-11-18

**Authors:** Hannah E. Goyne, Martin J. Cannon

**Affiliations:** ^1^Department of Pathology, University of Arkansas for Medical Sciences, Little Rock, AR, USA; ^2^Department of Microbiology and Immunology, University of Arkansas for Medical Sciences, Little Rock, AR, USA; ^3^Department of Obstetrics and Gynecology, University of Arkansas for Medical Sciences, Little Rock, AR, USA

**Keywords:** ovarian cancer, regulatory T cells, Th17 T cells, dendritic cells, indoleamine 2,3-dioxygenase

## Abstract

Clinical optimism for dendritic cell vaccination against ovarian cancer has been tempered by the knowledge that tumors avail themselves of multiple mechanisms of immune evasion, thus blunting the efficacy of therapeutic vaccination. Mechanisms of immune suppression include infiltration by regulatory T cells (Treg) and myeloid suppressor cell populations, expression of co-inhibitory receptors, and expression of indoleamine 2,3-dioxygenase (IDO). Expression of both B7-H1 and IDO are associated with differentiation and recruitment of Treg, and clinical studies have shown that each of these mechanisms correlates independently with increased morbidity and mortality in ovarian cancer patients. In sharp contrast, recent studies have indicated that Th17 cell infiltration in ovarian cancer correlates with improved patient outcomes and prolonged overall survival. Given that IDO plays a pivotal role in the balance between Treg and Th17 immunity, elucidation of the mechanisms that regulate IDO activity and immune suppression may lead to novel adjuvants to boost the clinical efficacy of dendritic cell vaccination against ovarian cancer and other malignancies.

## The Clinical Problem

Clinical studies have shown that the immune system plays an active and possible critical role in the pathogenesis of ovarian cancer, disease progression, and overall survival. Of the positive parameters, CD3 T cell infiltration has been associated with prolonged survival (1). A notable point from this investigation was that patients with significant T cell infiltration in their tumors were more likely to be optimally debulked during surgery provided an indication that T cells may limit regional spread of the disease. In contrast with these positive findings, the majority of studies have highlighted multiple mechanisms of immune suppression that correlate with poor patient outcomes in ovarian cancer.

Regulatory T cells (Treg) infiltration has been widely noted as a negative correlate of clinical outcomes for many malignancies, and ovarian cancer is no exception. Curiel and colleagues showed that Treg infiltration in ovarian cancer correlates with a poor prognosis and increased mortality (2). Other investigators have shown that high expression of Foxp3 (a transcription factor associated with a Treg phenotype) is an independent prognostic indicator for reduced survival (3), and that a high CD8/Treg ratio is associated with more favorable outcomes (4). Further mechanisms that contribute to the immunosuppressed state include expression of PD-L1 (B7-H1), which can promote T cell anergy and apoptosis through engagement of PD-1 expressed by effector T cells (5, 6) and expression of indoleamine 2,3-dioxygenase (IDO). Expression of both B7-H1 and IDO are associated with differentiation and recruitment of Treg (7–9), and clinical studies have shown that each of these mechanisms correlates independently with increased morbidity and mortality in ovarian cancer patients (10–12). Immune suppression in the tumor micro-environment is also likely to present a formidable barrier to the clinical efficacy of therapeutic tumor vaccination, including dendritic cell (DC) vaccination.

In sharp contrast with the evidence that Treg infiltration is associated with poor outcomes in ovarian cancer, Th17 T cell infiltration correlates with more favorable clinical outcomes (13). Furthermore, tumor-infiltrating Th17 cells were negatively associated with Treg infiltration, suggesting a reciprocal relationship between these subsets. These observations have led to the question of whether therapeutic benefit would accrue from induction or expansion of Th17 cells, either through DC vaccination, other types of tumor vaccines or adoptive immunotherapy (14, 15).

## Can Dendritic Cells Stimulate Th17 Responses Against Ovarian Cancer?

The tumor micro-environment can modify DC function through multiple mechanisms, usually resulting in inhibition of DC activation and maturation, and the induction of immunosuppressive DC and related myeloid cell populations (16). Tumor inhibition of DC function can also have an impact on therapeutic DC vaccines, indicating the need for DC vaccines with the capability to redirect T cell immunity from immune suppression to pro-inflammatory anti-tumor responses. Several lines of evidence have pointed to a crucial role for MAPK signaling pathways in regulation of pro-inflammatory versus tolerogenic or immunosuppressive DC function. Notably, Jackson and colleagues demonstrated that blockade of MEK 1/2 and ERK MAPK signaling restores tumor-mediated inhibition of DC function and promotes IL-12 production and Th1 T cell responses, whereas inhibition of p38 MAPK increases signal transduction through ERK 1/2 and blocks IL-12 production (17). In similar vein, p38 MAPK signaling in DC up-regulates IL-10 expression and induces tolerance in a mouse model of melanoma, resulting in suppression of anti-tumor T cell response, whereas inhibition of p38 signaling restored the ability of DC to stimulate T cell responses (18). The observation that p38 inhibition or MEK/ERK activation restores DC function in myeloma patients provides further evidence that p38 blockade may be of therapeutic benefit (19).

With respect to the balance between Treg versus Th17 immunity, studies in mice have shown that p38 inhibition attenuates Treg induction by DC and enhances the efficacy of DC vaccination and anti-tumor immunity (20), whereas blockade of the ERK pathway suppresses DC-driven Th17 responses (21). Collectively, these results suggest that preferential signaling though the ERK pathway may favor a switch from DC induction of Treg responses to Th17 differentiation and expansion.

In humans, treatment of ovarian tumor antigen-loaded, cytokine-matured DC with a combination of IL-15 and a p38 MAPK inhibitor offers potent synergy in antagonism of Treg induction and redirection toward Th17 responses that correlate with strong CD8^+^ CTL activation (22). Tumor antigen-specific CD4^+^ T cells secreted high levels of IL-17 and showed reduced expression of CTLA-4, PD-1, and Foxp3 following activation with IL-15/p38 inhibitor-treated DC. It was further shown that modulation of p38 MAPK signaling was associated with reduced expression of PD-L1 (B7-H1), loss of IDO activity, and increased phosphorylation of ERK1/2 MAPK. These observations afford an opportunity to develop innovative DC vaccination strategies to boost Th17 immunity in ovarian cancer patients.

## IDO and the Balance of Power between Treg and Th17 Immunity

Several lines of investigation have pointed to a pivotal role for IDO in directing Treg or Th17 responses in tumor immunity. In humans, IDO-expressing mature DC induce proliferation of CD4^+^CD25^+^Foxp3^+^ Treg (9) and parallel studies in mice have shown that IDO activates Treg and inhibits their conversion to Th17-like T cells (8, 23). The pathways involved in control of Treg/Th17 differentiation by IDO have hitherto been obscure, but recent studies have revealed a relationship between IDO function and the aryl hydrocarbon receptor (AhR) on DC and T cells. Binding of the AhR promotes the generation of Treg (24–26) and AhR ligand-specific interactions may control the balance between Treg and Th17 differentiation (27, 28). Remarkably, the tryptophan catabolite kynurenine produced by IDO is a natural ligand for AhR (29, 30), thus creating a mechanism by which IDO promotes generation of Treg.

The AhR is also expressed by DC, and is required for induction of IDO expression, thus creating a feedback loop via kynurenine that maintains DC regulatory function (31). DC from AhR^−/−^ mice lacked IDO and inhibited Treg development and promoted Th17 generation from naïve T cells. Addition of exogenous kynurenine restored the generation of Foxp3^+^ Treg and diminished Th17 differentiation, reinforcing the hypothesis that IDO, kynurenine, and AhR regulate the balance between Treg and Th17 immunity.

**Figure 1:**
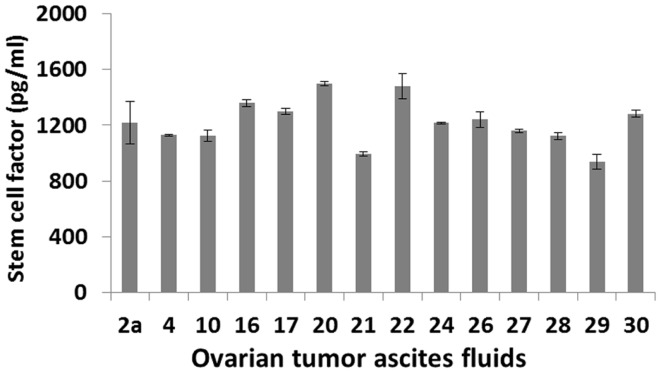
**SCF levels in primary ovarian tumor ascites fluids**. Samples from 14 patients with a confirmed diagnosis of epithelial ovarian cancer were tested by ELISA.

The mechanisms by which IDO expression is regulated in ovarian tumors and tumor-associated myeloid cells are largely unknown. One potential mechanism is that IDO activity may be driven by c-KIT signaling following binding of stem cell factor (SCF), which is secreted by ovarian tumors (32–34). This proposal is based in part on recent studies showing that IDO expression can be blocked by inhibitors of c-KIT or mTOR (downstream of the c-KIT-PI3K-AKT pathway), with resultant potentiation of anti-tumor T cell responses (35). Furthermore, siRNA knockdown of SCF or blockade of c-KIT can inhibit myeloid-derived suppressor cell expansion, Treg development and tumor angiogenesis, producing a synergistic therapeutic effect in combination with immunotherapy (36). Given that SCF is abundantly present in ovarian tumor ascites (Figure 1), these findings raise the possibility that similar mechanisms of immune regulation may prevail in ovarian cancer.

**Figure 2:**
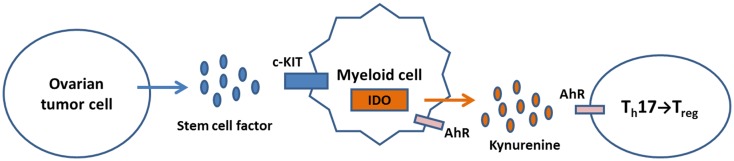
**A model for regulation of IDO expression and Treg/Th17 immunity in the ovarian tumor micro-environment**. Tumor production of SCF activates c-KIT signaling in TAM, leading to induction of IDO expression. The tryptophan catabolite kynurenine binds AhR on T cells, which shifts the Th17/Treg balance in favor of Treg generation. Kynurenine can also bind AhR on myeloid cells, further inducing IDO expression through a feedback loop.

Collectively, these observations allow formulation of an innovative model in which SCF binds c-KIT expressed by ovarian tumor cells or infiltrating myeloid cells, resulting in IDO expression. Kynurenine produced by IDO activity binds AhR on T cells and induces Treg differentiation (Figure 2). Drugs that block c-KIT signaling or IDO function may inhibit Treg recruitment and alleviate immune suppression by shifting the balance in favor of Th17 T cell differentiation and expansion.

## Innovative Strategies for Alleviation of IDO-Mediated Immune Suppression

Although the ability to manipulate the functional plasticity of DC to stimulate Th17 responses against ovarian tumor antigens represents a therapeutic opportunity, DC vaccination may not be clinically effective in the face of substantial barriers imposed by immune suppression in the tumor micro-environment. Direct depletion of tumor-associated Treg has often been the favored means of alleviating immune suppression in support of DC vaccination or other tumor vaccine strategies. Depletion of Treg activity may be achieved by treatment with low dose cyclophosphamide or denileukin diftitox (ONTAK) (37, 38). The ability of cyclophosphamide to inhibit Treg and boost anti-tumor immunity has been known for decades (39), and this drug is now widely used as an adjuvant to tumor vaccination in clinical trials. Recent studies have shown that cyclophosphamide promotes Th17 responses in cancer patients (40), lending further credence to its use as an adjuvant for DC vaccination against ovarian cancer.

A more favorable and durable approach may be to alleviate tumor-associated immune suppression through modulation of myeloid cell function and/or inhibition of IDO. If we accept the proposed model for regulation of IDO expression and Treg generation as a working hypothesis, we can infer that agents with the ability to inhibit c-KIT/PI3K/AKT/mTOR signaling or otherwise modify myeloid cell function may reduce IDO activity and inhibit recruitment of Treg in the ovarian tumor micro-environment. Our focus will be on drugs that are FDA-approved for other indications and have shown the ability to alleviate immune suppression and/or boost the efficacy of tumor vaccines or immune therapy in animal models. Promising candidates include imatinib mesylate, sunitinib, temsirolimus, and zoledronate, all of which have significant potential as adjuvants for DC vaccination against ovarian cancer.

Imatinib mesylate (Gleevec) binds BCR-ABL and c-KIT, and is an effective treatment for BCR-ABL^+^ chronic myeloid leukemia. More recent studies have shown that the therapeutic effect of imatinib could also be attributed to immune response, overcoming tumor-associated T cell tolerance and boosting vaccine efficacy (41). Imatinib also decreased Treg frequencies and enhanced anti-tumor immune responses to DC vaccination against imatinib-resistant BCR-ABL-negative lymphoma (42), and was subsequently shown to activate CD8^+^ T cells and induce Treg apoptosis in a gastrointestinal tumor model through c-KIT inhibition and diminished IDO expression (35).

With respect to ovarian cancer, KIT ligand (SCF) is anti-apoptotic and increases cisplatin resistance, whereas imatinib induces apoptosis (43). Although imatinib has shown minimal clinical benefit as a single agent in ovarian cancer (44, 45), it is well tolerated, and its ability to inhibit c-KIT and block IDO expression (35) suggests imatinib has potential to alleviate immune suppression as an adjuvant treatment for DC vaccination.

Sunitinib is an inhibitor of VEGFR, PDGFR, c-KIT, and Flt-3, and is FDA-approved for metastatic renal cell cancer. Sunitinib is currently being tested in over 300 clinical trials for cancer treatment (46), including ovarian cancer (47, 48). Numerous studies have shown that sunitinib can reduce myeloid suppressor cell accumulation and decrease Treg frequencies in animal models (49, 50) and in renal cell carcinoma patients (51, 52). This activity may at least in part be mediated through c-KIT and/or STAT3 signaling (49, 50). Sunitinib has been tested in combination with DC loaded with autologous total tumor RNA in a recently completed phase II clinical trial (NCT00678119), and a new phase III trial of DC vaccination for renal cell carcinoma following first-line treatment with sunitinib has recently been initiated (NCT01582672). No results have been reported to date for either trial.

Axitinib, a related tyrosine kinase inhibitor that blocks multiple targets, including c-KIT, may also have potential as adjuvant therapy for DC vaccination. Axitinib was approved by the FDA in 2012, as a second line treatment for advanced renal cell carcinoma.

Rapamycin (an mTOR inhibitor) is well known for its ability to suppress T cell responses, but it also has potential as an anticancer agent through inhibition of HIF-1, HIF-2, and VEGF. mTOR is a downstream component of the KIT-PI3K-AKT pathway, and rapamycin can reproduce imatinib-mediated reduction of IDO (35). Temsirolimus (a rapamycin analog) was FDA-approved for the treatment of renal cancer in 2007, and is first-line treatment for patients with metastatic disease. Remarkably, temsirolimus can enhance the efficacy of tumor vaccines (53), suggesting that it does not share the immunosuppressive properties of rapamycin, and may have value as an adjuvant for DC vaccination.

Although they don’t act as inhibitors of c-KIT or downstream signaling, amino-bisphosphonates may also have potential as adjuvant treatments, by virtue of their ability to modify myeloid cell function. Zoledronic acid is a matrix metalloprotease inhibitor that blocks myeloid-derived suppressor cell expansion (54) and induces a pro-inflammatory shift in macrophage function, favoring M1 polarization over the pro-tumor M2 phenotype. Zoledronate can reduce expression of VEGF and IL-10 and increase production of type-1 cytokines such as IFNγ (55, 56). It is not known whether amino-bisphosphonates have any influence on IDO expression.

## Direct Inhibition of IDO

While it would appear reasonable to block IDO function directly, rather than use signal transduction inhibitors that might lead to off-target effects and toxicity, agents that inhibit IDO activity are few and far between. 1-Methyl tryptophan has seen extensive experimental use as a competitive blocker of IDO function (57, 58), but its clinical use has been limited. There are currently two trials of DC vaccination combined with 1-methyl tryptophan listed by ClinicalTrials.gov: a phase I/II trial of adenovirus p53-transduced DC vaccine with 1-methyl tryptophan for treatment of recurrent/stage IV breast cancer (NCT01042535, sponsored by the National Cancer Institute), and a phase II trial of Sipuleucel-T (Provenge^®^, Dendreon Corp.) with 1-methyl tryptophan for treatment of metastatic castration-resistant prostate cancer (NCT01560923, sponsored by the Masonic Cancer Center, University of Minnesota).

A second agent, INCB24360, developed by Incyte Corporation, is being tested in a phase II trial versus tamoxifen for patients with ovarian, primary peritoneal, or fallopian tube cancer and suffering biochemical recurrence, i.e., with CA125 at least twice the upper limit of normal (NCT01685255). INCB24360 is also being tested in a randomized phase II trial of ipilimumab plus INCB24360 or placebo for metastatic melanoma (NCT01604889). Should INCB24360 demonstrate efficacy in inhibition of IDO activity in the clinical setting, it may have considerable value as an adjuvant treatment for DC vaccination against ovarian cancer and other malignancies.

## Conclusion

Elucidation of the multiple facets of immune regulation in the ovarian tumor micro-environment has sharpened the appreciation that there are formidable barriers to therapeutic DC vaccination, but has also raised the prospect for mechanism-based interventions. The observations that Treg infiltration or Th17 infiltration respectively correlate with either diminished or improved overall survival strongly indicate that blockade of Treg activity or stimulation of Th17 responses could similarly result in improved clinical outcomes. DC vaccine strategies that bias tumor-specific T cell responses toward a Th17 phenotype should be tested in clinical trials, preferably in advanced stage ovarian cancer patients that have completed surgery and chemotherapy and have minimal disease at the time of DC vaccination. The goal should be to prevent disease recurrence or progression, rather than to use DC vaccination as a salvage therapy in patients with significant tumor burden.

Although DC vaccination designed to boost Th17 immunity represents a step forward, adjuvant treatments that alleviate tumor-associated immune suppression are probably essential for any prospect of clinical success. IDO expression by ovarian tumor cells or infiltrating myeloid cells arguably forms one of the cornerstones of immune regulation in ovarian cancer, and it is no surprise that high IDO expression is associated with diminished overall survival. Drugs that block IDO expression or activity may tip the Treg/Th17 balance in favor of anti-tumor immunity, and several intriguing possibilities that are either FDA-approved for other indications or are currently in clinical trials have been considered. Targeting of the IDO/kynurenine/AhR regulatory pathway may also be an innovative approach, e.g., through the use of AhR antagonists such as resveratrol.

Other treatments that can have an impact on immune regulation in ovarian cancer should also be considered, either as stand-alone therapy or in combination with DC vaccination. Ipilimumab targets the T cell inhibitory molecule CTLA-4, which is highly expressed by Treg, and is FDA-approved for treatment of metastatic melanoma. There are many clinical trials in progress for ipilimumab in treatment of other malignancies, including ovarian cancer, but results have been variable and often discouraging. The prospects might be better for anti-PD-1 antibodies or anti-PD-L1 antibodies, for which promising results have been reported from clinical trials. Given that PD-L1 (B7-H1) expression is associated with decreased overall survival in ovarian cancer, blockade of PD-L1, or PD-1 may be an attractive option. Collectively, though, it is our opinion that the weight of evidence points to IDO as the focal target for immunological intervention in support of DC vaccination against ovarian cancer.

## Author Contributions

Hannah E. Goyne and Martin J. Cannon contributed equally to the preparation and writing of this manuscript.

## Conflict of Interest Statement

Martin J. Cannon is a founder of DCV Technologies, Inc., a biotechnology company dedicated to the clinical development of dendritic cell vaccines for the treatment of cancer. The authors declare no other conflicts of interest.
